# Scrub Typhus Presenting With Meningoencephalitis and Multiorgan Dysfunction Syndrome

**DOI:** 10.7759/cureus.107041

**Published:** 2026-04-14

**Authors:** Adithya Mani, Sharan Prasaanth, Muthukumarasamy Thiyagarajan

**Affiliations:** 1 Internal Medicine, Coimbatore Medical College, Coimbatore, IND; 2 Medicine, Stanley Medical College, Chennai, IND

**Keywords:** doxycycline, multiorgan dysfunction syndrome, orientia tsutsugamushi, scrub typhus meningitis, vasculitis

## Abstract

Scrub typhus is an acute febrile illness endemic in India and remains an important yet often underdiagnosed cause of severe systemic infection. It is caused by *Orientia tsutsugamushi* and is characterized by endothelial injury leading to widespread vasculitis. We report the case of a 62-year-old male with a history of chronic alcohol use and no known comorbidities, who presented with a short history of fever, myalgia, arthralgia, mild confusion, and gait difficulty. The illness rapidly progressed to scrub typhus septicemia complicated by pulmonary edema, pleural effusion, meningoencephalitis, acute renal and hepatic injury, culminating in multiorgan dysfunction syndrome. He was treated with doxycycline and ceftriaxone along with supportive care, resulting in clinical improvement. This case highlights the wide spectrum of clinical manifestations of scrub typhus and underscores the importance of early diagnosis and timely intervention to reduce morbidity and mortality.

## Introduction

Acute febrile illness (AFI) is a common clinical presentation in tropical regions such as India, where infections such as dengue, malaria, enteric fever, and scrub typhus contribute significantly to morbidity and mortality [1]. Scrub typhus, caused by *Orientia tsutsugamushi* and transmitted by infected chigger mites, is an important and increasingly recognized cause of acute undifferentiated fever [2]. It is endemic within the “tsutsugamushi triangle,” including parts of Asia and northern Australia, and has shown a re-emergence in recent years, particularly in rural and agricultural regions. The disease is characterized by endothelial infection leading to vasculitis and perivascular inflammation, resulting in multiorgan involvement [3]. Following dissemination, vascular injury and increased permeability contribute to complications affecting the lungs, liver, kidneys, heart, and central nervous system. Clinically, scrub typhus presents with acute fever, headache, and myalgia, often accompanied by gastrointestinal symptoms [4]. An eschar, though characteristic, may not always be present [5]. Severe manifestations include acute respiratory distress syndrome, acute kidney injury, hepatitis, myocarditis, and shock. Neurological involvement such as meningoencephalitis is increasingly recognized and may present with altered sensorium or seizures [6]. The differential diagnosis includes other common causes of AFI, such as dengue, malaria, leptospirosis, and enteric fever, which share overlapping clinical and laboratory features. Diagnosis is typically based on serological tests such as the IgM enzyme-linked immunosorbent assay (ELISA), while molecular methods such as PCR offer early detection where available. Scrub typhus is a treatable condition, with doxycycline and azithromycin being the mainstay of therapy [7]. Early diagnosis and prompt treatment are crucial, as delayed management can lead to severe complications, including multiorgan dysfunction syndrome and increased mortality. We report a case of scrub typhus complicated by meningoencephalitis and multiorgan dysfunction to highlight the diverse clinical spectrum and the importance of early recognition and management.

## Case presentation

Presenting symptoms

A 62-year-old male with no known comorbidities was referred from a community hospital with a provisional diagnosis of AFI with thrombocytopenia and acute kidney disease. He presented with a three-day history of high-grade fever associated with arthralgia, myalgia, difficulty in walking, bilateral lower limb swelling, and mild confusion. There was no history of recent travel, rash, or bleeding manifestations. The patient had a history of chronic alcohol consumption and was unable to recall any episode suggestive of an insect bite. On general examination, the patient was febrile, and initial vital parameters were stable.

During the course of hospitalization, he developed progressive respiratory distress. Respiratory system examination revealed bilateral basal crepitations, and subsequent evaluation confirmed pulmonary edema with pleural effusion. Neurologically, the patient exhibited worsening sensorium with neck rigidity and Kernig's sign and was diagnosed with acute meningoencephalitis. Abdominal examination was unremarkable except for bilateral pedal edema, while laboratory investigations revealed deranged renal and hepatic function parameters.

Laboratory findings

**Figure 1:**
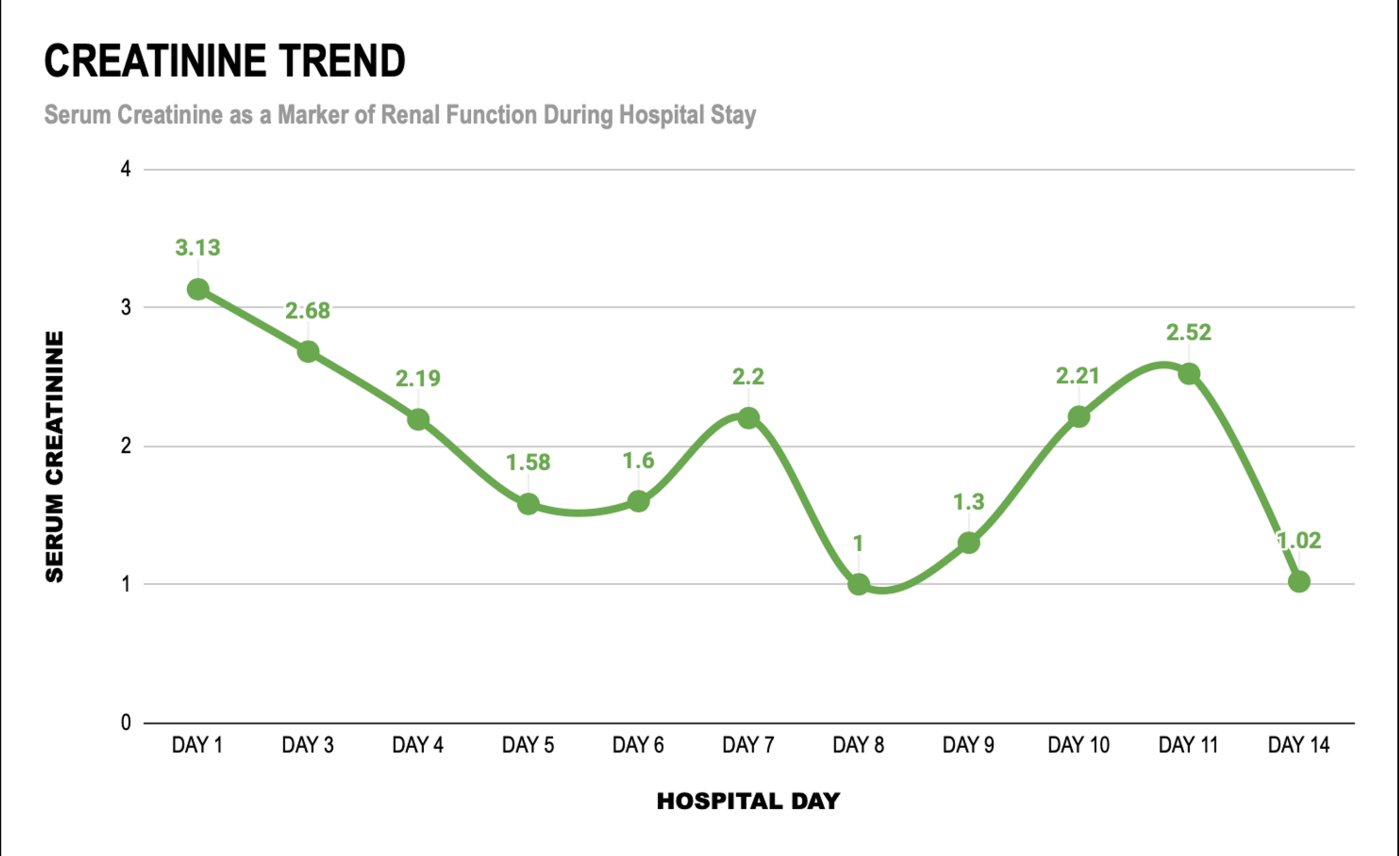
Clinicobiochemical trend of serum creatinine during hospital stay

Initial complete blood count revealed normal hemoglobin levels with thrombocytopenia; however, the patient developed anemia over the course of the illness. Platelet counts showed fluctuation on serial monitoring. Serum lactate dehydrogenase levels were markedly elevated (>1200 U/L). Renal function tests were significantly deranged, consistent with acute kidney injury with uremia (135 mg/dL) (Figure 1). Electrolyte analysis demonstrated hypernatremia (152 mEq/L) and high-normal potassium levels (5.30 mEq/L) initially. Liver function tests revealed hepatic involvement with elevated transaminases, including an aspartate aminotransferase level of 122 U/L and an alkaline phosphatase level of 425 U/L on Day 3 (Table 1). Coagulation studies showed prolonged prothrombin time and activated partial thromboplastin time, with an international normalized ratio greater than 1. Blood and sputum cultures yielded no bacterial growth. As part of the evaluation for AFI, investigations for other tropical infections, including dengue and leptospirosis, were negative. Cerebrospinal fluid (CSF) analysis did not reveal any abnormalities. 

**Table 1:** Laboratory investigations

Parameters	Reference range	Day 1	Day 2	Day 3	Day 10	Day 11	Day 14
Hemoglobin (g/dL)	13.0-17.0	12.3	11.9	13	7.9	8.4	9
Platelets (×10³/µL)	150-450	27	18	53	25	66	119
Urea (mg/dL)	15-30	135	179	160	130	163	16
Creatinine (mg/dL)	0.6-1.2	3.13	2.68	2.19	2.21	2.52	1.02
Serum bilirubin (mg/dL)	0.3-1.2	3.55	2.6	2.18	0.76	0.72	0.3
Alkaline phosphatase (IU/L)	30-120	329	314	425	148	173	95

Imaging studies

**Figure 2:**
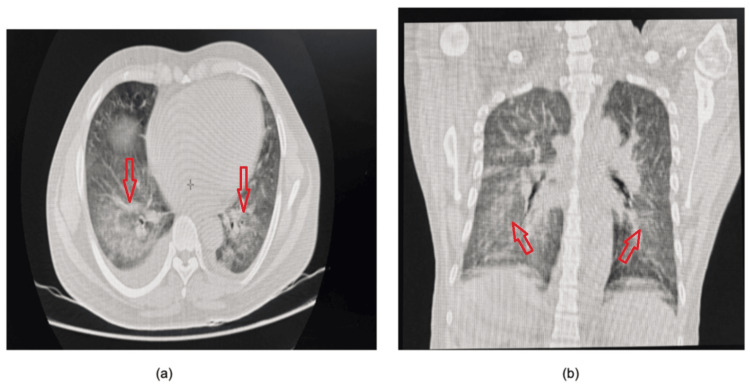
(a) Axial and (b) coronal lung window showing perihilar ground-glass opacities.

Chest radiography revealed bilateral basal homogeneous opacities. Further evaluation was performed with computed tomography (CT) of the chest and ultrasonography. Ultrasonography demonstrated minimal bilateral pleural effusions. CT of the chest revealed mild bilateral pleural effusions with ground-glass opacities involving the right upper lobe and bilateral lower lobes, along with patchy air-space opacities (Figure 2). The absence of significant cardiomegaly on imaging, along with a normal two-dimensional echocardiogram showing normal cardiac structure and function, suggested a non-cardiogenic cause of the pulmonary edema and pleural effusion [8]. Neuroimaging in the form of a non-contrast CT scan of the brain was performed and did not reveal any intracranial abnormalities.

Diagnosis and management 

Based on the clinical presentation, the presence of a pathognomonic eschar located in the left lumbar region of the abdomen, and serological confirmation by scrub typhus IgM ELISA [9], the patient was diagnosed with scrub typhus. He had already been initiated on empirical doxycycline therapy at a dose of 100 mg twice daily, which was continued as doxycycline remains the drug of choice for scrub typhus. In addition, intravenous ceftriaxone was administered along with comprehensive supportive care. During hospitalization, the patient developed worsening respiratory distress and altered sensorium, necessitating endotracheal intubation and mechanical ventilation [10]. Supportive management for pulmonary edema and pleural effusion included oxygen therapy, ventilatory support, and adjunctive measures. The clinical course was further complicated by acute kidney injury with refractory hyperkalemia, for which he underwent hemodialysis via right femoral vein catheterization using the modified Seldinger technique [11]. Symptomatic and supportive treatment included intravenous antibiotics, analgesics, antiemetics, proton pump inhibitors, nebulization, bronchodilators, probiotics, vitamins, cholagogues, and other supportive measures as indicated. The patient demonstrated a gradual clinical response, with resolution of fever and progressive improvement in neurological, respiratory, renal, and hepatic parameters.

## Discussion

Scrub typhus, caused by *Orientia tsutsugamushi*, is an important cause of AFI in endemic regions such as India and can progress to severe complications, including encephalitis and multiorgan dysfunction syndrome (MODS). Delayed diagnosis contributes significantly to morbidity and mortality due to its nonspecific presentation.

Neurological involvement is a recognized manifestation of scrub typhus. Although CSF abnormalities are commonly described, normal CSF findings do not exclude central nervous system involvement. In our patient, clinical signs of meningeal irritation, including neck stiffness and a positive Kernig’s sign, were present despite unremarkable CSF analysis. The prompt resolution of these signs following doxycycline therapy strongly supports a diagnosis of scrub typhus-related meningitis [12] and highlights the clinicobiochemical dissociation observed in this condition.

MODS in scrub typhus results from widespread endothelial dysfunction and systemic inflammation. Our patient demonstrated involvement of multiple organ systems, including hepatic, renal, respiratory, and hematological systems, while notably lacking cardiac involvement, reflecting the heterogeneous pattern of organ dysfunction reported in severe scrub typhus.

**Figure 3:**
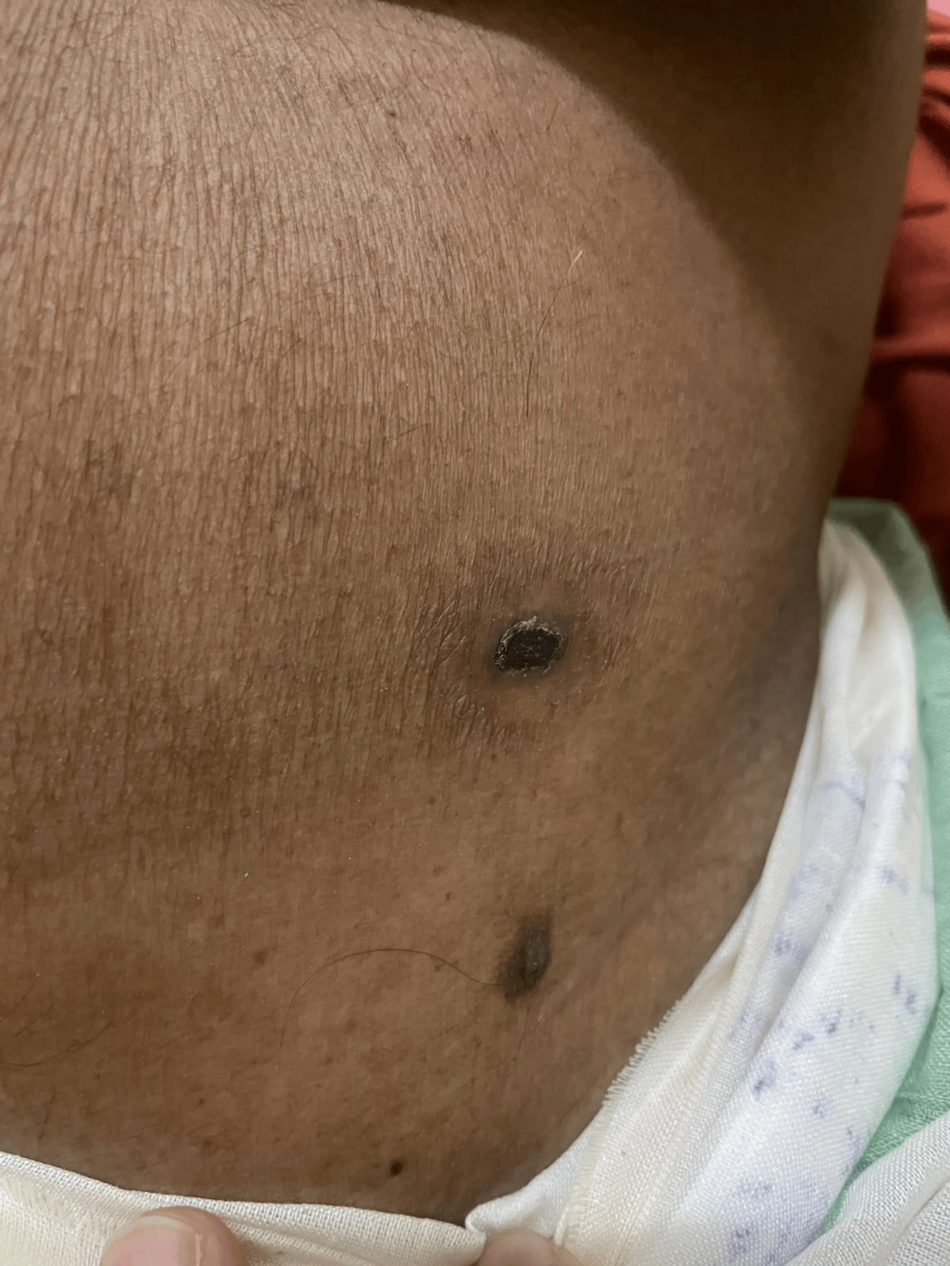
Characteristic eschar in the left lumbar region

The identification of a characteristic eschar [13] (Figure 3) over the left lumbar region provided a crucial and pathognomonic diagnostic clue in this case, significantly strengthening the clinical suspicion of scrub typhus in the setting of nonspecific systemic and neurological features. Careful and thorough physical examination is essential, as eschars may be easily overlooked or absent, particularly when located in concealed areas such as the axilla, groin, or lumbar region. Recognition of this lesion can expedite diagnosis and prompt initiation of appropriate therapy, thereby preventing progression to severe disease.

Doxycycline remains the treatment of choice for scrub typhus and is associated with rapid clinical improvement. In this case, doxycycline was administered along with ceftriaxone due to initial diagnostic uncertainty in a patient presenting with meningoencephalitis. The rapid defervescence and neurological recovery following doxycycline initiation confirmed scrub typhus as the primary etiology.

This case underscores the importance of considering scrub typhus in patients with AFI, meningeal signs, and MODS, even in the absence of classical CSF findings. Early recognition and timely treatment can be lifesaving in this potentially fatal yet readily treatable disease.

## Conclusions

Scrub typhus should be considered an important differential diagnosis in patients presenting with AFI and unexplained multiorgan dysfunction in endemic regions. This case underscores its potential to present with severe complications such as meningoencephalitis and multiorgan dysfunction syndrome, which can be life-threatening if not recognized early. The absence of classic signs may delay diagnosis, making a high index of suspicion essential. Early initiation of doxycycline resulted in marked clinical recovery in our patient, emphasizing that timely recognition and treatment can significantly reduce morbidity and mortality. Greater clinical awareness of atypical and severe manifestations of scrub typhus is crucial for improving patient outcomes.
